# Primer Extension Mutagenesis Powered by Selective Rolling Circle Amplification

**DOI:** 10.1371/journal.pone.0031817

**Published:** 2012-02-15

**Authors:** Tuomas Huovinen, Eeva-Christine Brockmann, Sultana Akter, Susan Perez-Gamarra, Jani Ylä-Pelto, Yuan Liu, Urpo Lamminmäki

**Affiliations:** Department of Biochemistry and Food Chemistry, University of Turku, Turku, Finland; Universita' di Milano, Italy

## Abstract

Primer extension mutagenesis is a popular tool to create libraries for *in vitro* evolution experiments. Here we describe a further improvement of the method described by T.A. Kunkel using uracil-containing single-stranded DNA as the template for the primer extension by additional uracil-DNA glycosylase treatment and rolling circle amplification (RCA) steps. It is shown that removal of uracil bases from the template leads to selective amplification of the nascently synthesized circular DNA strand carrying the desired mutations by phi29 DNA polymerase. Selective RCA (sRCA) of the DNA heteroduplex formed in Kunkel's mutagenesis increases the mutagenesis efficiency from 50% close to 100% and the number of transformants 300-fold without notable diversity bias. We also observed that both the mutated and the wild-type DNA were present in at least one third of the cells transformed directly with Kunkel's heteroduplex. In contrast, the cells transformed with sRCA product contained only mutated DNA. In sRCA, the complex cell-based selection for the mutant strand is replaced with the more controllable enzyme-based selection and less DNA is needed for library creation. Construction of a gene library of ten billion members is demonstrated with the described method with 240 nanograms of DNA as starting material.

## Introduction

Large randomized gene libraries are an invaluable tool for the up-to-date protein engineering. In many applications the most appropriate diversity is achieved by targeted randomization of selected residues or domains of interest. Typically, targeting of mutations relies on a synthetic pool of oligonucleotides carrying the desired mutations that are incorporated into the target gene by *de novo* gene assembly or by using wild type gene as template. In the latter case, the template-hybridized primer is extended at moderate temperature, for example with T4 or T7 DNA polymerase, or at elevated temperature, with thermostable DNA polymerases as in PCR, to finalize the incorporation.

One of the early primer extension methods used uracil-containing circular single-stranded DNA (ss(U)DNA) template. No phenotypic selection was needed as the nascently synthesized mutant strand contained no uracil and was thus favoured in bacterial propagation resulting in reported 50% mutagenesis efficiency [1]. This method, termed Kunkel mutagenesis, has subsequently been modified to be suitable also for double-stranded (ds) DNA. This has been achieved by additional nitrocellulose filtering steps for alkali denatured template ds(U)DNA [2], by utilizing a second oligo to destroy a unique restriction enzyme site for template sequence removal [3] and by digesting the heteroduplex ds(U)DNA with DpnI, which removes methylated DNA template [4]. However, the reported numbers of obtained transformants with ds(U)DNA templates in these experiments are only from hundreds to tens of thousands colony forming units.

In contrast to the ds(U)DNA experiments, libraries with more than billion members have been established by using ss(U)DNA templates [5], [6]. The ss(U)DNA-method has been further extended by mutating several regions of the template gene simultaneously. This strategy requires that the template DNA sequence is modified to contain stop codons at the sites of mutagenesis to prevent the translation of the wild type protein, as the wild type template is passed to the final product at high proportions cutting down the overall efficiency of mutagenesis [5], [6]. Generation of the billion-member libraries requires tens of micrograms of affinity purified single-stranded template DNA.

In the past ten years, phi29 DNA polymerase has been extensively used in various applications including cell-free cloning [7], whole genome amplification [8] and *in situ* genotyping [9]. The alluring features of the phi29 DNA polymerase are the high processivity and strong strand displacement activity resulting in efficient isothermal DNA amplification [10]. Dean et al. (2001) reported that the input circular DNA was amplified 10 000 times by rolling circle mechanism (RCA) in a randomly primed, phi29 DNA polymerase driven, reaction [11].

In this study, we introduce a novel strategy to reduce the template background involved in Kunkel mutagenesis and increase the amount of transformable DNA for large scale library production. A common feature to the family B DNA polymerases, including the phi29 DNA polymerase, is that the presence of an abasic site in the template DNA is a strong stalling signal [12]. We treated the Kunkel mutagenesis product with uracil-DNA glycosylase (UDG), which catalyses the excision of the uracil bases from the template, and amplified the product with phi29 DNA polymerase.

In the three separate experimental case studies we demonstrate that UDG treatment of the Kunkel heteroduplex leads to selective amplification of only the intact mutated strand by phi29 DNA polymerase. No template was observed after the selective amplification. In contrast, many clones that were obtained after direct transformation of the Kunkel product were still wild type. In addition, we observed that the wild type gene coexisted with the mutant in a significant portion of the Kunkel transformants. Large libraries of high mutant frequency are achieved with the described method by replacing the complex host-dependent selection for the mutated strand with the well-defined enzyme-dependent selection in primer extension mutagenesis.

## Results

### Principle

In Kunkel mutagenesis the template is prepared by transforming phagemid carrying the gene of interest into an *ung^−^dut^−^ E. coli* host, like CJ236 or BW313. These strains are deficient in the enzyme dUTP pyrophosphatase (*dut^−^*) resulting in an increased incorporation of uracil in place of thymine in DNA [13]. The incorporated uracil is not removed from the DNA due to an inactivating point mutation in uracil-DNA glycosylase (*ung^−^*) [14], [15]. The uridylated template is gained in single-stranded form by extracting the phagemid DNA from filamentous phage particles released from infected host cells.

The oligonucleotide or pool of oligonucleotides containing the desired changes is annealed to the ss(U)DNA template. The 5′- and 3′-regions of the randomized oligonucleotide are complementary to the template and target the oligonucleotide to the intended site of mutagenesis. After extension and ligation a covalently closed circular double-stranded heteroduplex DNA (ccc-ds(U)DNA) is formed, in which the newly *in vitro* synthesized DNA contains the desired mutations - but no uracil. The mutated strand should have a strong selective advantage over the template when the heteroduplex DNA is propagated in *ung^+^ dut^+^ E. coli* host, because uracil-containing DNA is biologically inactivated in the host [1].

**Figure 1:**
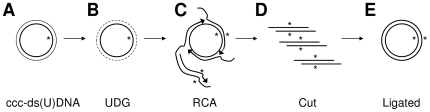
Selective rolling circle amplification (RCA) flowchart. The heteroduplex, ccc-ds(U)DNA formed in Kunkel mutagenesis (A), is treated with UDG (B) and subsequently amplified with RCA (C) using random hexamers as primers. The resulting DNA concatemer is cut to plasmid-sized units (D) and re-circularized by self-ligation (E) for host transformation.

**Figure 2:**
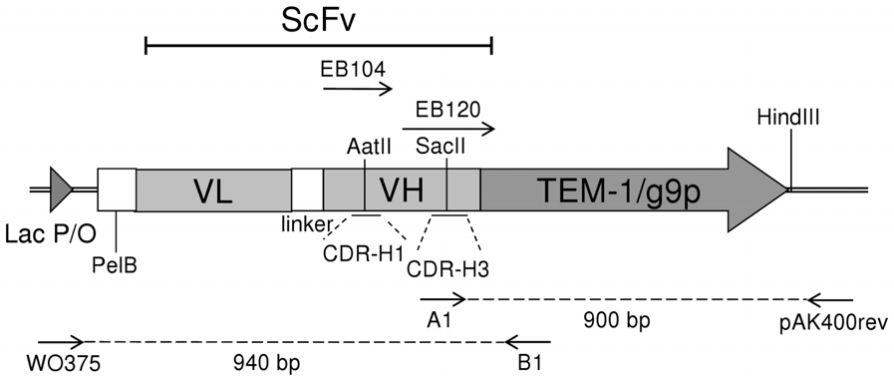
Target of mutagenesis. In the CDR-H1 loop mutagenesis and VH-gene incorporation scFv gene was fused to gene 9-protein (g9p) of the filamentous phage. In the CDR-H3 loop mutagenesis scFv was fused in-frame to β-lactamase (TEM-1). EB104 was the mutagenesis primer in the CDR-H1 and EB120 in CDR-H3 mutagenesis, respectively. The primer pairs WO375-B1 and A1-pAK400rev were used in the analysis of transformants of the CDR-H3 mutagenesis. (ScFv) Single-chain variable fragment, (VL) immunoglobulin variable light domain gene, (VH) immunoglobulin variable heavy domain gene, (PelB) signal sequence for periplasmic excretion and (Lac P/O) Lac promoter.

For selective amplification of the mutant strand, the ccc-ds(U)DNA is treated with UDG and amplified with phi29 DNA polymerase in the presence of random primers. Due to the abasic sites created by the UDG treatment, phi29 DNA polymerase amplifies selectively the intact mutated strand producing multiple copies of the phagemid. The resulting DNA concatemer is subsequently monomerized with a single-cut-per-phagemid restriction enzyme digestion and self-ligation. Fig. 1 presents a schematic overview of the presented technique termed selective RCA (sRCA). Our target of mutagenesis with the most important restriction enzyme and primer hybridization sites is illustrated in Figure 2.

### Increased mutagenesis efficiency with sRCA

The first study of sRCA was carried out by randomizing CDR-H1 loop of a single-chain variable fragment (scFv) antibody gene. A 51 nt long primer, termed EB104, contained two randomized codon positions flanked by 23 and 19 template-complementary nucleotides in the 5′- and 3′-ends, respectively. EB104 primer was hybridized to the ss(U)DNA template and further extended by Kunkel mutagenesis method. A sample of the product was UDG-treated and selectively amplified by RCA.

A successful mutagenesis destroys an AatII restriction enzyme site present in the template. Consequently, mutagenesis frequencies of the directly transformed and selectively amplified sample sets were compared by restriction digestion of 10 single clone DNA preparations. The mutagenesis frequency was increased from 30% with direct Kunkel mutagenesis to 100% with selective RCA. In fact, one of the three AatII-sensitive clones from Kunkel mutagenesis was completely digested with the enzyme, whereas the other two clones were partially digested. Sequencing of these clones revealed that the two partially digested clones contained also the template sequence. Accordingly, in the totally cleaved sample only the altered genotype was present.

To obtain more mutants for sequencing from the Kunkel sample, the cells transformed with the Kunkel mutagenesis product were harvested and a DNA miniprep was prepared. The DNA preparation was digested with AatII, which linearizes unmutated plasmids at the CDR-H1 region and a sample was re-transformed. After this refinement, 9/10 Kunkel samples were mutants based on sequencing (Table 1).

**Table 1:** Diversity based on sequencing in CDR-H1 loop mutagenesis and VH-gene incorporation experiments.

Target of analysis	CDR-H1 loop		VH-gene incorporation	
Theoretical diversity	30		14	
Treatment	K	K+U+R	K+U	K+U+R
Template	1^a^	0	3	0

	mutated gene variants			
1 copy	7	7	6	5
2 copies	1	1	3	4
3 copies	0	0	1	1
4 copies	0	0	0	1
Unreadable	0	1	2	0
**Total, N**	**10**	**10**	**20**	**20**
Unique	8	8	10	11

K: Kunkel, U: UDG and R: RCA.

a AatII digestion was used to eliminate unmutated template clones. Before AatII digestion 7/10 were template clones according to restriction enzyme analysis.

The first and second randomized position of the primer EB104 was a mix of six and five codons, respectively, bringing the total diversity to 30 variants. The primer was synthesized using trinucleotide phosphoramidites as building blocks. In both the Kunkel and RCA sets 7/10 clones were correctly mutated. Obviously, the primer synthesis was defective as two clones in sRCA set contained identical trinucleotide deletions in the randomized positions. One clone from the Kunkel mutagenesis had two extra bases at the randomization site and another clone in close vicinity. Taking them into account, there were 8 unique sequences in both the Kunkel and the RCA sample group. The groups consisted of different mutants, apart from one clone that was present in both, covering together 12/30 designed variants.

### Construction of large libraries via PCR product incorporation

To explore the achievable library size and applicability to incorporate PCR products, a new scFv gene library was built-up by VL shuffling: 14 VH gene variants were amplified by asymmetric PCR and incorporated as a pool into a readily available scFv ss(U)DNA library containing 10^8^ VL variants [16]. Transformation of 240 ng of the UDG-treated Kunkel reaction directly to SS320 cells resulted in a library of 3×10^7^ cfu. Selective amplification of the same amount of UDG-treated Kunkel with RCA yielded 12 µg of affinity purified re-circularized DNA. 1×10^10^ cfu were obtained by transforming half of the 12 µg which is over 300 times more than without selective RCA.

Sequencing twenty single clones of the UDG-treated Kunkel and sRCA reactions showed that the distribution of incorporated VH gene variants was more or less similar. Ten and eleven unique variants were found in the Kunkel and sRCA sample groups, respectively (Table 1). The identified VH gene pools were partially overlapping as 7/14 sequences were present in both, 3/14 only in the Kunkel and 4/14 only in the sRCA sample. All of the sequenced VL genes differed from each other, which is an expected observation due to the large VL diversity of 1×10^8^ variants.

### CDR-H3 loop mutagenesis of ScFv-β-lactamase fusion gene

#### Experimental set-up

Next, an easy assay system was developed to analyze the mutagenesis outcome of different treatments by plating transformed cells on selective agar plates. The target for mutagenesis was the CDR-H3 loop region of a scFv antibody gene fused in-frame to TEM-1 β-lactamase gene. There were three stop codons in the template sequence at the site of mutagenesis which is restored to open reading frame by the oligonucleotide. The oligonucleotide mix EB120 was used for this study containing seven NNN codons flanked by 28 hybridizing nt in the 5′-end and 25 hybridizing nt in the 3′-end. In theory, if the distribution of all codons (including the three stop codons) is nonbiased 78% of the mutated clones should have open reading frame and thus, result in an ampicillin resistant clone. This complex randomized oligonucleotide mix was chosen for the study to test the system in a library set-up with theoretical translated diversity of 10^9^ members compared to the limited diversities incorporated in the earlier examples.

EB120 was incorporated by Kunkel mutagenesis and the effect of UDG treatment and RCA were systematically studied. To test the importance of UDG treatment during RCA both UDG treated and nontreated heteroduplex were amplified with phi29 DNA polymerase. 10 ng DNA samples of all treatments were transformed to XL-1 Blue cells and the cells were plated on agar plates containing only chloramphenicol (cm) and the combination of chloramphenicol and ampicillin (cm & amp). The cm-plates selected for all clones carrying the phagemid pEB07-scFv and the combination cm & amp for the mutated clones with the restored reading frame.

#### UDG treatment is essential for selective amplification

**Figure 3:**
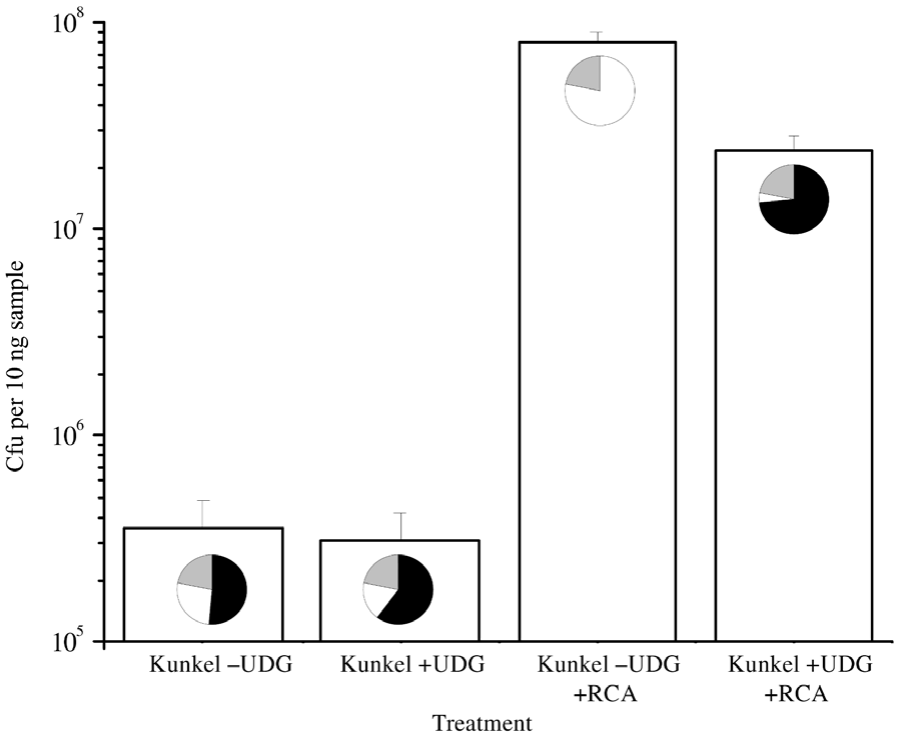
The effect of UDG treatment on Kunkel and selective rolling circle amplification (RCA) mutagenesis. CDR-H3 region was altered by Kunkel mutagenesis and further treated with +/− UDG and amplified with RCA. The mutant yield was studied by plating transformed samples. Successful CDR-H3 primer incorporation in scFv-β-lactamase gene resulted in ampicillin resistant clones (pie chart, black sector). The template clones were sensitive to ampicillin (pie chart, white sector). In theory, 22% of the mutated clones with 7× NNN codons are not ampicillin resistant due to primer-born STOP-codons (pie chart, grey sector).

The mutagenesis efficiency was calculated as the ratio of cm & amp-resistant (ampR) to cm-resistant clones (cmR). Direct transformation of the Kunkel heteroduplex resulted in 51.3±7.6% ampR/cmR in the functional mutagenesis assay based on three independent experiments (Fig. 3). UDG treatment improved the mutagenesis efficiency to 60.4±18.0% and after selective RCA functional mutagenesis efficiencies of 65.1±4.7% and 73.6±11.2% were achieved depending on whether exoresistant or unmodified random primers were used in the amplification reaction. Surprisingly, RCA of the heteroduplex without UDG treatment decreased the mutagenesis efficiency to 0.1±0.01%.

In addition to increasing the portion of mutated clones in the population, selective RCA increased the total number of transformants (Fig. 3). Transformation of 10 ng affinity purified heteroduplex directly yielded 3.6±1.3×10^5^ cfu and after UDG treatment 3.1±1.1×10^5^ cfu, respectively. Based on sample transformations, a library of 8.1±1.7×10^7^ cfu, i.e. 260 times more transformants per ng of source DNA, would be obtained by transforming all DNA amplified from the 10 ng sample of UDG-treated heteroduplex by selective RCA. Taking into account the synergistic effect of the higher selectivity for mutants and the larger library size the absolute number of correctly mutated clones increased from 1.9×10^5^ mutants gained by direct transformation of the 10 ng UDG-treated heteroduplex to 6.0×10^7^ mutants with sRCA exceeding the unamplified yields over 300-fold.

**Figure 4:**
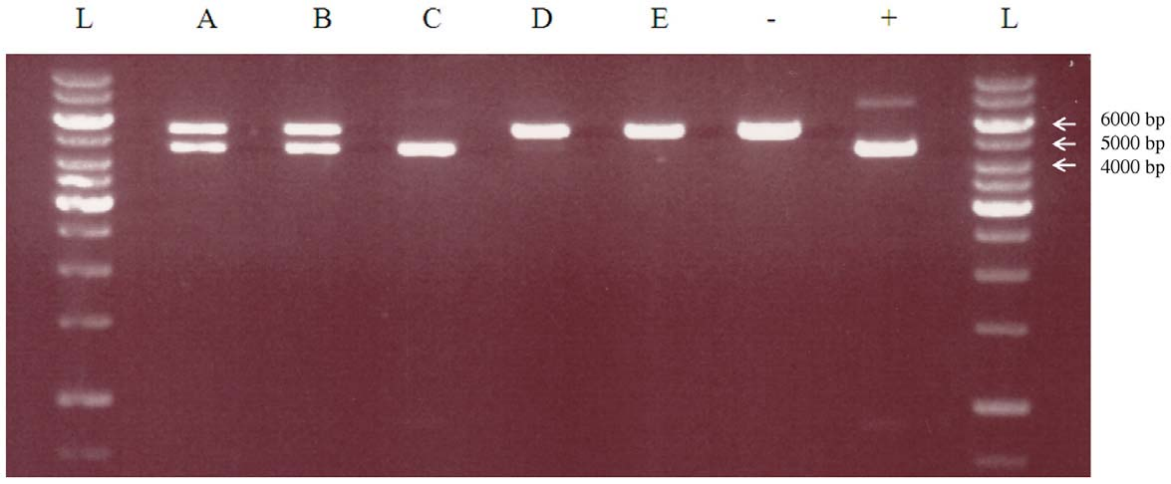
Digestion of 1 µg mutated phagemid DNA library pools with HindIII and SacII. (A) Kunkel −UDG, (B) Kunkel + UDG, (C) Kunkel −UDG +RCA, (D) Kunkel +UDG +RCA with exoresistant random primers, (E) Kunkel +UDG +RCA with normal random primers, (−) SacII resistant control, (+) SacII sensitive control, (L) 1 kb Fermentas DNA Ladder.

Another qualitative confirmation of the mutagenesis efficiency was obtained by digesting 1 µg of library DNA extracted from overnight grown cells transformed with the Kunkel, UDG-treated Kunkel and selective RCA samples (Fig. 4). HindIII site is outside the target gene and hence present in all phagemids, whereas SacII site is present only in the wild type gene. Therefore, the unmutated phagemids are cleaved to 4506 bp and 868 bp fragments and the mutated phagemids are linearized to 5374 bp fragments. The 868 bp band is visible on lane C and the SacII-sensitive control lane with similar intensity. It was also visible on lanes A and B in visual inspection under UV-light. The lower intensity of the 868 bp band is a natural consequence of the five-fold smaller fragment size, as one mole of 868 bp DNA fragments have only 20% of the amount of intercalated EtBr compared to one mole of 4506 bp DNA fragments. According to the clearly visible 4.5 kb and 5.4 kb fragments both mutated and unmutated phagemids were present in the Kunkel and UDG-treated Kunkel libraries (A,B). Without UDG treatment amplified sample did not have any visible amount of mutated phagemids (C), sharply contrasted by the UDG-treated sRCA samples in which the only visible fragment indicated mutated DNA (D,E). This confirms the previous finding, that UDG treatment is essential to amplify selectively only the mutated strand of the Kunkel heteroduplex and that the selective amplification results in nearly complete loss of the unmutated template.

#### Elimination of template-mutant coexistence by sRCA

Due to the variation and inaccuracy caused by the cell transformations, dilutions, platings and the presence of possible STOP-codons in the randomized region, mutagenesis efficiency was re-analysed using colony PCR. As described before, a scFv gene amplicon is resistant to cleavage with SacII if mutagenesis has been successful. In a sample group of 20 colonies originating from the Kunkel sample and picked from cm-plates 8/20 amplicons were perfectly cleaved by SacII, 8/20 were cleavage-resistant and the remaining 4/20 partially digested (Table 2). In the group originating from the UDG-treated Kunkel sample the result was identical except that there were 3/20 partially digested and 9/20 cleavage-resistant amplicons. In contrast, all analysed transformants, 20/20, originating from the UDG-treated and selectively amplified library primed with unmodified random hexamers were resistant to SacII digestion indicating 100% mutagenesis efficiency. When exoresistant hexamers were used in RCA, 2/20 clones were non-mutated and 18/20 mutated. No partial digestion was observed among the selectively amplified transformants. The size of all PCR products was uniform indicating that neither Kunkel nor sRCA caused severe DNA rearrangements.

**Table 2:** Colony PCR screen of clones created with primer extension mutagenesis of CDR-H3 loop.

		Analysis I			Analysis II
Treatment	Random hexamers	SacII resistant	SacII-sensitive	Partial digestion with SacII	Presence of template DNA
K	none	8/20	8/20	4/20	11/34
K+U	none	9/20	8/20	3/20	16/34
K+U+R	exoresistant	18/20	2/20	0/20	0/31
K+U+R	normal	20/20	0/20	0/20	0/34

Analysis I: colonies from cm-plates (all grow) amplified with WO375 & B1 (all amplified) and digested with SacII. Analysis II: colonies from cm & amp-plates (mutants grow) amplified with A1 & pAK400rev (wild type specific). K: Kunkel, U: UDG, R: RCA, cm: chloramphenicol and amp: ampicillin.

Some of the samples in the SacII digestion analysis were classified as partially digested, because a control amplicon derived from the wild type template was totally digested with SacII in the same conditions. Partial digestion indicates that both mutated and unmutated phagemids had propagated in those clones. To re-verify the presence of double templates in the Kunkel and UDG-treated samples, we decided to test a new set of colonies from the cm & amp-plates. In this screen there is no possibility for overlapping wild type and mutant colonies as the wild type does not grow on the plate.

A pair of primers was used in which the forward primer hybridized only to the wild type template DNA and the reverse primer outside the mutated region. In this way, smaller amounts of template DNA could be detected than by digesting the amplicon. In the case of Kunkel heteroduplex 11/34 clones yielded PCR product indicating that the unmutated template DNA was still present in 32% of the clones considered to be mutated (Table 2). The prevalence of the unmutated template among the UDG-treated Kunkel transformants was similar to the previous, whereas no PCR product was observed among the sRCA transformants.

#### Follow-up of the double sequence clones

**Figure 5:**
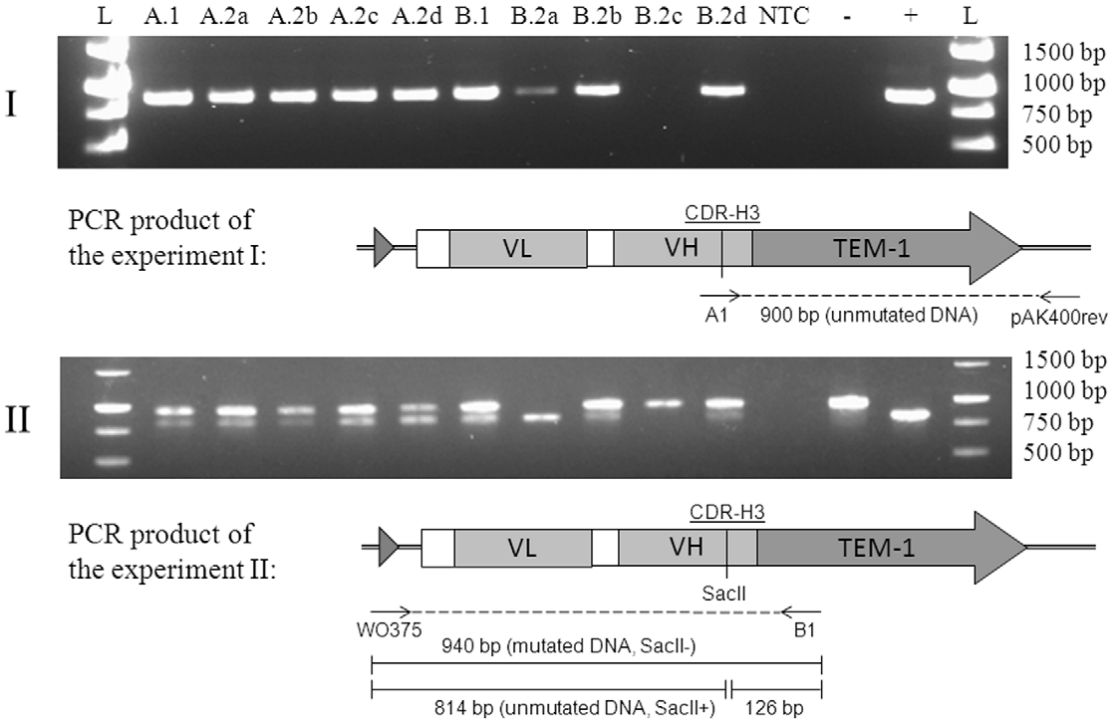
Colony PCR analysis of the progeny of two primary clones (A.1 and B.1) originating from the Kunkel mutagenesis of CDR-H3 loop. (I) PCR with wild-type-template- specific primer A1 and pAK400rev. 900 bp product is formed, if template is present. (II) SacII digestion analysis of PCR products (940 bp) primed with WO375 and B1 that hybridize outside the CDR-H3 loop. Both wild-type and mutated DNA is amplified, but only the wild-type DNA is cut to 814 bp and 126 bp fragments. (A.2a–d) Daughter colonies of A.1; (B.2a–d) Daughter colonies of B.1; (−) SacII resistant control, (+) SacII sensitive control, (L) 1 kb Fermentas DNA Ladder bands 1500–500 bp.

In theory, a β-lactamase-excreting clone could shield other nonresistant overlapping clones from the antibiotic activity of ampicillin. Therefore, two putative double-template ampR-clones were streaked on cm-plates and grown overnight for clonal separation. Cm-plates without amp were chosen for growing the pure cultures in order to avoid selective pressure for the ampR-phagemids corresponding better to the conditions in mutagenesis experiments without phenotypic selection. Four daughter colonies from each parent were screened for the presence of unmutated DNA. Based on wild type specific PCR and SacII digestion analysis, unmutated DNA was still present in 4/4 and 3/4 daughter colonies (Fig. 5). When the template-specific PCR was continued from 26 to 31 cycles, a band emerged also from the clone B.2c that was considered to be fully mutated after 26 cycles.

The proportions of mutated-to-unmutated phagemids diverged from each other among the daughter cells (Fig. 5). For example, the daughter colony A.2d cells harboured equal amounts of mutated and unmutated DNA, whereas the other three colonies of the same family contained more of the mutated cleavage-resistant DNA according to the SacII digestion test. The progeny of the other colony, B, is even more heterogeneous: no visible amount of SacII-resistant mutated DNA was observed in B.2a cells and in B.2c all visible DNA was cleaved with SacII. Eight new daughter colonies from both clones were streaked from the cm-plates again to amp-plate for the third generation of colonies. All of them were, however, still resistant to ampicillin indicating that none of them had totally lost the mutant phenotype despite the significant variation that was observed in the mutated-to-unmutated DNA ratio between the daughter cells.

## Discussion

Kunkel mutagenesis relies on the biological selection mechanism in the host cell favoring propagation of the nascently synthesized DNA strand of desired genotype over the uridylated wild type strand. Kunkel reasoned that the uridylated template strand is biologically inactivated by native UDG and apurinic/apyrimidinic (AP) endonucleases leading to strand breaks, since the introduced abasic sites in the ssDNA genome of phi ×174 phage and M13 had lethal consequences in transfection assays [17]–[19].

Evidently, the inactivation of the uridylated DNA is efficient in ssDNA, but the fate of the uridylated strand present in a double-stranded DNA heteroduplex is more complex. In general, the uracil encountered in the *E. coli* genome is removed by the uracil-mediated base excision DNA repair (BER) process. The native UDG initiates the BER process followed by cleavage of the phosphodiester bond on the 5′-side of the abasic site by AP endonuclease [20]. After removal of the deoxyribose 5′-phosphate (including the surrounding nucleotides in some experiments [21]) the gap is filled based on the template sequence [20]. Thus, the uracil frequency in DNA and the expression levels of BER-enzymes in the host cell may play a vital role in the biological selection, as too little amount of uracil may lead to complete repair of the template causing background to arise.

In a cell also other mechanisms than the BER pathway have an influence on the integrity of the strands. For example, the methyl-directed mismatch correction system [22] favors the naturally methylated, albeit uridylated, strand and may eliminate the desired mutations from the unmethylated synthesized strand.

According to our results, uridylation of the template and the UDG-treatment of the uridylated template *in vitro* clearly increase the occurrence of the altered genotype among the transformants, but neither of them are adequate discriminators to totally eliminate the template strand. This was observed by digesting the library DNA preparations, colony PCR experiments and sequencing. In many cases co-existence of the wild type gene with the mutant does not cause trouble, but in studies where the desired mutation creates a positive phenotype, for example restoration of the LacZα complementation [2], [4], the researchers remain unaware of the presence of the template background.

In sRCA, the discrimination in favor of the mutant strand and against the uracil-excised template strand is based on the substrate specificity of the phi29 DNA polymerase providing full control over the process. The heteroduplex DNA is homogenized by sRCA and therefore, the complex cell repair systems do not have an influence on the outcome. In addition, sRCA of a Kunkel reaction increases the DNA yields and, consequently, the number of transformants up to 300-fold in our experiments. Despite the amplification, no noteworthy bias in diversity was observed in the CDR-H1 loop mutagenesis and VH-gene incorporation studies. Naturally, new genotypes are not generated in the selective amplification phase, but as less than 1 in 100 molecules of circular supercoiled plasmid can be successfully transformed into host by electroporation [23], the only way to increase the number of different recombinant clones in a library, is to produce more DNA in an unbiased way.

Interestingly, without UDG treatment the ccc-ds(U)DNA is very efficiently amplified and the mutation frequency is decreased from the original 51% to 0.1%. Therefore, an incomplete UDG treatment causes dramatic losses in mutant recovery. This observation may be explained either by phi29 DNA polymerase favoring the uracil-containing strand over the nascently synthesized strand or by the Kunkel reaction being incomplete due to failing extension or ligation.

It is known that phi29 DNA polymerase is able to extend uracil-containing template and catalyse the incorporation of dUTP opposite dA, with only 2-fold lower efficiency than dTTP [24], but currently, no biochemical evidence shows that the DNA polymerase would actually favor the U-strand over the other. More probably, there may be a large excess of the intact wild type ss(U)DNA present in a Kunkel reaction due to failing hybridization, extension or ligation serving as the main substrate for amplification. The amplification potential of this background is effectively eradicated by the UDG treatment.

Recently, we have introduced a new method to re-circularize the RCA product by replacing the single-cut-per-phagemid restriction digestion and self-ligation with Cre/*LoxP* recombination. After inserting the *loxP* recombination site of bacteriophage P1 Cre recombinase into the target phagemid, the concatemer generated in sRCA is reduced to plasmid-sized units by simple Cre recombinase addition [25]. Furthermore, selective RCA may be performed in error-prone conditions [26] supplementing the site-directed mutations incorporated during Kunkel's reaction with random mutations generated in RCA. To conclude, selective RCA is a novel tool to increase the total yield and frequency of desired changes in primer extension mutagenesis. Starting with few hundred nanograms of Kunkel's heteroduplex DNA, billion-member gene libraries can be achieved without severe diversity bias.

## Materials and Methods

### Kunkel mutagenesis of CDR-H1 loop

The mutagenesis oligo EB104 (5′-CA GCC TCC GGA TTT ACG TTC TCC [X3] TAC [X4] ATG CAC TGG GTC CGT CAG G-3′) was synthesized using trinucleotide phosphoramidites as building blocks at University of Kuopio, Department of Pharmaceutical Chemistry, Finland in a procedure similar to reported earlier [27]. There were two randomized codon positions in EB104. [X3] consisted of a pool of codons TCT, AAC, GAC, CGT, ACT, GGT (16.7% each) and [X4]: TAC, GGT, GCT, TGG, TCT (20% each), respectively. EB104 was phosphorylated in a 20 µl reaction containing 5 µM oligo, 1 mM ATP, 5 mM DTT, 70 mM Tris-HCl (pH 7.6), 10 mM MgCl_2_ and 20 U T4 polynucleotide kinase (NEB, Ipswich, USA) for 1 h at 37°C.

Phage stock of the pEB91-scFv phagemid was prepared from *E. coli* CJ236 strain (NEB, Ipswich, USA) with standard methods [6] with the exception that the CJ236 growth medium was supplemented with 6 µg/ml uridine (Sigma-Aldrich, St. Louis, USA). The ss(U)DNA was extracted with Qiaspin M13 Kit (Qiagen, Hamburg, Germany) according to manufacturer's instructions.

The phosphorylated primer EB104 was annealed to 1 µg of single-stranded pEB91-scFv [16] at 4∶1 molar ratio in 1× T4 DNA ligase buffer (NEB, Ipswich, USA). The primer hybridization was done following the program: 90°C for 2 min, 70°C for 2 min, 50°C for 5 min, 20°C for 5 min, 4°C for 5 min. Reactions were set on ice for 10 min.

The following components were added to the 15 µl annealing reaction while keeping on ice: 1 µl 25 mM MgCl_2_, 2.5 µl 10 mM ATP, 2 µl 100 mM DTT, 1 µl 25 mM dNTP, 3.5 U T7 DNA polymerase (NEB, Ipswich, USA) and 3 Weiss Units T4 DNA ligase (NEB, Ipswich, USA). The reaction was incubated at room temperature for 3 h to extend the primer. The reaction containing the completed Kunkel heteroduplex was heat inactivated at 75°C for 20 min.

### Selective RCA of the CDR-H1 loop Kunkel product

2 µl of the heteroduplex reaction was treated with UDG in a 10 µl reaction containing 20 mM Tris-HCl (pH 8.0), 1 mM EDTA, 1 mM DTT and 1 U UDG (NEB, Ipswich, USA) for 15 min at 37°C. The reaction was purified with Qiagen PCR purification Kit (Qiagen, Hamburg, Germany) and eluted into 50 µl of 10 mM Tris-HCl (pH 8.5). 5 µl UDG-treated heteroduplex was amplified in a 20 µl reaction with 1 mM dNTP, 50 µM random hexamers (Fermentas, St. Leon-Rot, Germany), 1× phi 29 DNA polymerase buffer containing 50 mM Tris-HCl (pH 7.5), 10 mM MgCl_2_, 10 mM (NH_4_)SO_4_, 4 mM DTT, 100 ng/ml BSA and 10 U Phi29 DNA polymerase (Fermentas, St. Leon-Rot, Germany). The reactions were incubated overnight at 30°C and inactivated at 70°C for 10 min. The amplified DNA was digested with 20 U XhoI (Fermentas, St. Leon-Rot, Germany) for 2 h to cut the DNA concatemer to monomer units, purified with Qiagen PCR purification kit and eluted into 50 µl 10 mM Tris-HCl (pH 8.5). 2 µl purified XhoI-digested DNA was re-circularized in a 20 µl reaction volume with 1× T4 DNA Ligase buffer containing 40 mM Tris-HCl (pH 7.8), 10 mM MgCl_2_, 10 mM DTT and 0.5 mM ATP. Reaction was supplemented with 10 U T4 DNA Ligase (Fermentas, St. Leon-Rot, Germany) and incubated at room temperature for 2 h. Ligation was inactivated at 65°C for 10 min.

DNA samples were electroporated into *Escherichia coli* XL-1 Blue (Agilent Technologies, Santa Clara, USA) and dilutions plated on LA containing 25 µg/ml chloramphenicol (cm), 10 µg/ml tetracycline (tet) and 0.5% glucose (Glc). Plates were incubated overnight at 37°C. DNA was extracted from single colonies and samples were sequenced. All sequencing in this study was performed by Sequencing Service at the Center of Biotechnology (Turku, Finland). The sequenced scFv-genes were deposited in Genbank under accession numbers JQ041909-JQ041965.

Due to the low efficiency in Kunkel mutagenesis, 2 µl of the DNA miniprep prepared from 5 ml overnight culture of the cells transformed with the Kunkel product was digested with 10 U AatII (Fermentas, St. Leon-Rot, Germany) overnight at 37°C in the manufacturer's recommended buffer to linearize the unmutated template clones. A sample of the digested DNA was re-transformed to obtain more mutants for sequencing.

### VH gene mutagenesis

Asymmetric PCR products of 14 VH genes originating from the pEB91-scFv universal library were prepared and used in a Kunkel-type primer extension reaction as described in [16]. The formed heteroduplex was treated with 10 U UDG (NEB, Ipswich, UK) at 37°C for 1 h and purified with Qiagen PCR purification kit to 50 µl 10 mM Tris-HCl (pH 8.5). 2 µl sample (240 ng i.e. 4×10^10^ molecules) was amplified in 200 µl phi29 DNA polymerase reaction buffer containing 1 mM dNTP's, 50 µM random primers (Fermentas, St. Leon-Rot, Germany), 4 mM DTT, 0.25 U Inorganic pyrophosphatase (Fermentas, St. Leon-Rot, Germany) and 100 U Phi29 DNA polymerase (Fermentas, St. Leon-Rot, Germany). Pyrophosphatase was added to prevent the accumulation of the inhibitory pyrophosphate in RCA [11]. The reactions were incubated overnight at 30°C and heat inactivated at 70°C for 10 min.

The DNA concatemer produced in RCA by Phi29 DNA polymerase was digested with 200 U HindIII to single-plasmid sized units in a 1 ml reaction volume overnight at 37°C and purified with miniprep kit columns (Qiagen, Hamburg, Germany) in the following way. The 1 ml digest was mixed with 5 ml binding buffer, applied in a 10 ml TERUMO-syringe (Terumo Corp., Tokyo, Japan), which was fastened tightly in a miniprep column. 3 ml of the digest per column was pressed through and, after loading, the purification was continued according to manufacturer's instructions with final 100 µl elution volume in 10 mM Tris-HCl (pH 8.5).

The HindIII-digested DNA was self-ligated overnight at 16°C by using 5 ng DNA/µl with 0.05 U/µl T4 DNA Ligase in the manufacturer's (Fermentas, St. Leon-Rot, Germany) recommended buffer containing 40 mM Tris-HCl (pH 7.8), 10 mM MgCl_2_, 10 mM DTT and 0.5 mM ATP. Concentration and purification of the ligation was carried out to 50 µl volume with the syringe-method described above. Samples of the UDG-treated and selectively amplified re-circularized RCA library were transformed to XL-1 Blue cells. DNA was extracted from single colonies and samples sequenced.

For large scale transformation, 240 ng of the affinity purified UDG-treated DNA (six transformations, 40 ng DNA and 70 µl cells/cuvette) and 6 µg of the re-circularized RCA product (25 transformations, 240 ng and 70 µl cells/cuvette) were transformed to fresh electrocompetent *E. coli* MC1061 F′ cells similar to SS320 [6] with Bio-Rad Genepulser (Bio-Rad, Hercules, USA) with settings 200 Ω, 1.25 kV, 25 µF. After recovery in 24 ml (UDG-treated) and 100 ml SOC (RCA) at 37°C shaken at 100 rpm for 1 h, samples were diluted and plated on LA (25 µg/ml cm, 10 µg/ml tet and 0.5% glc) for counting transformation efficiency.

### CDR-H3 loop mutagenesis of ScFv-β-lactamase fusion gene

Vector pEB07 was constructed by replacing the myc tag and p9 gene sequences in vector pEB91 [16] with TEM-1 β-lactamase gene using SacI and HindIII sites. ScFv[NONSTOP] gene was purchased from Entelechon (Regensburg, Germany) and cloned to the SfiI sites of the vector pEB07 in frame with the β-lactamase. ScFv[STOP] contains SacII site and three stop codons at the CDR-H3 loop region and it was constructed with Kunkel mutagenesis from the ScFv[NONSTOP]-construct that lacks the SacII site and the stop codons. Functionality of the vectors pEB07-ScFv[STOP] and pEB07-ScFv[NONSTOP] was tested by plating transformed cells on cm- and cm & amp-plates. The [STOP]-construct was growing only on the cm-plate and the [NONSTOP]-construct on both plates. pEB07-ScFv[STOP] is referred to by the name pEB07-ScFv, if not otherwise stated.

The ss(U)DNA of the pEB07-scFv phagemid was prepared as with pEB91-ScFv. The mutagenesis oligo EB120 (5′-CTAGTGTACCCTGACCCCAGTCCATAGC NNNNNNNNNNNNNNNNNNNNN ACGAGCACAGTAGTAGACAGCCGTG-3′) was purchased from TAG Copenhagen (Copenhagen, Denmark). The oligo was phosphorylated, hybridized to ss(U)DNA and extended as described in Kunkel mutagenesis for CDR-H1 loop. The obtained heteroduplex was either directly transformed to *E. coli XL-1* Blue or subjected to an UDG treatment, in which, 1 µg Kunkel heteroduplex was treated in 1× UDG reaction buffer (NEB, Ipswich, USA) containing 20 mM Tris-HCl (pH 8.0), 1 mM EDTA, 1 mM DTT and 10 U UDG (NEB, Ipswich, USA) for 1 h at 37°C in a total volume of 25 µl.

A control sample without UDG treatment was incubated in the same conditions as the UDG treated sample with the exception that the UDG was replaced with H_2_O. Reactions were purified with Qiagen PCR purification Kit and eluted into 50 µl of 10 mM Tris-HCl (pH 8.5). DNA concentrations were determined with Nanodrop (Nanodrop Technologies, Wilmington, USA).

RCA of 10 ng DNA samples of the UDG treated and nontreated reactions were performed in a 20 µl reaction volume as described in VH gene incorporation. Either exoresistant random primers or normal random hexamers (both from Fermentas, St. Leon-Rot, Germany) were used in the reaction. DNA samples were digested for 2 h at 37°C with 20 U HindIII (Fermentas, St. Leon-Rot, Germany) in a 50 µl reaction supplemented with the manufacturer's recommended reaction buffer containing 10 mM Tris-HCl (pH 8.5), 10 mM MgCl_2_, 100 mM KCl and 0.1 mg/ml BSA. The reactions were purified with Qiagen PCR purification kit and eluted into 50 µl 10 mM Tris-HCl (pH 8.5). DNA concentrations were determined with Nanodrop (Thermo Fisher Scientific, Waltham, USA).

1 µg samples of the purified HindIII-digested DNA were re-circularized in a 200 µl reaction volume with 25 U T4 DNA Ligase (Fermentas, St. Leon-Rot, Germany) as described earlier with VH gene mutagenesis. Ligations were purified with Qiagen PCR purification Kit, eluted in 50 µl Tris-HCl (pH 8.5) and DNA concentrations were determined with Nanodrop (Thermo Fisher Scientific, Waltham, USA).

### Colony PCR analysis of CDR-H3 loop mutants

10 ng DNA samples from CDR-H3 loop mutagenesis of pEB07-scFv were electroporated into XL-1 Blue cells and recovered in 1 ml SOC for 1 h. 100 µl dilutions were spread on LA containing 25 µg/ml cm and 100 µM isopropyl β-D-1-thiogalactopyranoside (IPTG), and to plates containing 100 µg/ml amp in addition to cm and IPTG. Plates were incubated overnight at 37°C and the colonies counted.

For colony PCR analysis, colonies were picked from both cm- and cm & amp-transformation plates in 20 µl H2O. The colonies from cm & amp-plate were re-streaked on cm-plates for the analysis of the next generation daughter colonies. The suspended colonies were heated at 98°C for 10 min, spinned 4000 rpm for 20 min and 1 µl supernatant was used as a template in PCR with a total 10 µl volume. PCR was performed with Phire Hot Start II DNA polymerase (Thermo Fisher Scientific, Waltham, USA) according to manufacturer's instructions.

Two separate PCR reactions were done for each clone. The presence of DNA was controlled with WO375-B1 primer pair. These primers hybridize outside the scFv-gene and amplify both mutated and unmutated DNA. Those samples that did not yield product with WO375-B1 were omitted from analysis. The presence of unmutated DNA was analysed with A1-pAK400rev primer pair. Primer A1 hybridizes to the CDR-H3 loop region of the wild type template and primer pAK400rev hybridizes outside the scFv-gene. Cycling conditions were: 1) 98°C for 30 s, 2) 98°C for 5 s, 3) 62°C (A1-pAK400rev) or 61°C (WO375-B1) and 4) 72°C for 20 s. Steps 2)–4) were repeated 30× and final extension was at 72°C for 1 min. The re-streaked cm & amp-mutants were PCR-amplified with the A1-pAK400rev pair from the secondary cm-plates with both 26 and 31 cycles.

### SacII digestion analysis of CDR-H3 region mutants

The presence of unmutated DNA was also analysed from cm-plate clones by digesting 2 µl of the WO375-B1 amplicons with 1 U SacII (NEB) for 4 h at 37°C in the manufacturer's recommended buffer. In addition, a phagemid pEB07-scFv[STOP] containing the SacII site was amplified with the same WO375-B1 primers and used as a positive control to assess the completeness of the SacII-digestions. pEB07-scFv[NONSTOP] lacking the SacII site was used as a negative control. All samples were run on 1% agarose gel for analysis. The cleavage resistant amplicon was 940 bp and after complete cleavage 814 bp and 126 bp fragments were formed.

In addition to single clones, library DNA preparations of the CDR-H3 mutagenesis reactions were prepared and analysed in the following way. After 1 h recovery in 1 ml SOC medium, transformed cells were diluted in 4 ml SB (Super broth: 30 g tryptone, 20 g yeast extract, 10 g MOPS per 1 l, pH 7.0) and supplemented with 25 µg/ml cm. Cultures were grown overnight at 37°C, 300 rpm and the DNA was extracted with Qiagen miniprep kit (Qiagen, Hamburg, Germany) according to manufacturer's instructions. Library DNA concentrations were determined with Nanodrop (Thermo Fisher Scientific, Waltham, USA). 1 µg DNA samples were first digested with SacII (Promega, Madison, Wisconsin, USA) in a 10 µl volume at 37°C o/n and then diluted to 20 µl volume for HindIII (Fermentas, St. Leon-Rot, Germany) digestion at 37°C for 4 h. 200 ng digested DNA samples were run on 1% agarose gel for analysis.

## References

[1] Kunkel T (1985). Rapid and efficient site-specific mutagenesis without phenotypic selection.. Proc Natl Acad Sci U S A.

[2] Jung R, Scott M, Oliveira L, Nielsen N (1992). A simple and efficient method for the oligodeoxyribonucleotide-directed mutagenesis of double-stranded plasmid DNA.. Gene.

[3] Markvardsen P, Lassen S, Borchert V, Clausen I (1995). Uracil-USE, an improved method for site-directed mutagenesis on double-stranded plasmid DNA.. Biotechniques.

[4] Li F, Liu S, Mullins J (1999). Site-directed mutagenesis using uracil-containing double-stranded DNA templates and DpnI digestion.. Biotechniques.

[5] Sidhu S, Li B, Chen Y, Fellouse F, Eigenbrot C (2004). Phage-displayed antibody libraries of synthetic heavy chain complementarity determining regions.. J Mol Biol.

[6] Sidhu S, Lowman H, Cunningham B, Wells J (2000). Phage display for selection of novel binding peptides.. Methods Enzymol.

[7] Hutchison CA, Smith HO, Pfannkoch C, Venter JC (2005). Cell-free cloning using phi29 DNA polymerase.. Proc Natl Acad Sci U S A.

[8] Dean F, Hosono S, Fang L, Wu X, Faruqi A (2002). Comprehensive human genome amplification using multiple displacement amplification.. Proc Natl Acad Sci U S A.

[9] Larsson C, Koch J, Nygren A, Janssen G, Raap AK (2004). In situ genotyping individual DNA molecules by target-primed rolling-circle amplification of padlock probes.. Nat Methods.

[10] Blanco L, Bernad A, Lázaro JM, Martín G, Garmendia C (1989). Highly efficient DNA synthesis by the phage phi 29 DNA polymerase. Symmetrical mode of DNA replication.. J Biol Chem.

[11] Dean F, Nelson J, Giesler T, Lasken R (2001). Rapid amplification of plasmid and phage DNA using Phi 29 DNA polymerase and multiply-primed rolling circle amplification.. Genome Res.

[12] Sagher D, Strauss B (1985). Abasic sites from cytosine as termination signals for DNA synthesis.. Nucleic Acids Res.

[13] Tye B, Lehman I (1977). Excision repair of uracil incorporated in DNA as a result of a defect in dUTPase.. J Mol Biol.

[14] Warner H, Duncan B, Garrett C, Neuhard J (1981). Synthesis and metabolism of uracil-containing deoxyribonucleic acid in Escherichia coli.. J Bacteriol.

[15] Lari S, Chen C, Vertéssy B, Morré J, Bennett S (2006). Quantitative determination of uracil residues in Escherichia coli DNA: Contribution of ung, dug, and dut genes to uracil avoidance.. DNA Repair (Amst).

[16] Brockmann EC, Akter S, Savukoski T, Huovinen T, Lehmusvuori A (2011). Synthetic single-framework antibody library integrated with rapid affinity maturation by VL shuffling.. Protein Eng Des Sel.

[17] Schaaper RM, Loeb LA (1981). Depurination causes mutations in SOS-induced cells.. Proc Natl Acad Sci U S A.

[18] Baas PD, van Teeffelen HA, Teertstra WR, Jansz HS, Veeneman GH (1980). Restoration of the biological activity of in vitro synthesized phi X DNA by transfection of ung- spheroplasts or dUTPase treatment.. FEBS Lett.

[19] Kunkel TA (1984). Mutational specificity of depurination.. Proc Natl Acad Sci U S A.

[20] Dianov G, Lindahl T (1994). Reconstitution of the DNA base excision-repair pathway.. Curr Biol.

[21] Sandigursky M, Freyer GA, Franklin WA (1998). The post-incision steps of the DNA base excision repair pathway in Escherichia coli: studies with a closed circular DNA substrate containing a single U:G base pair.. Nucleic Acids Res.

[22] Glickman BW, Radman M (1980). Escherichia coli mutator mutants deficient in methylation-instructed DNA mismatch correction.. Proc Natl Acad Sci U S A.

[23] Dower WJ, Miller JF, Ragsdale CW (1988). High efficiency transformation of E. coli by high voltage electroporation.. Nucleic Acids Res.

[24] Serrano-Heras G, Bravo A, Salas M (2008). Phage phi29 protein p56 prevents viral DNA replication impairment caused by uracil excision activity of uracil-DNA glycosylase.. Proc Natl Acad Sci U S A.

[25] Huovinen T, Julin M, Sanmark H, Lamminmäki U (2011). Enhanced error-prone RCA mutagenesis by concatemer resolution.. Plasmid.

[26] Fujii R, Kitaoka M, Hayashi K (2004). One-step random mutagenesis by error-prone rolling circle amplification.. Nucleic Acids Res.

[27] Yagodkin A, Azhayev A, Roivainen J, Antopolsky M, Kayushin A (2007). Improved synthesis of trinucleotide phosphoramidites and generation of randomized oligonucleotide libraries.. Nucleosides Nucleotides Nucleic Acids.

